# Polymorphism of carbonic acid (H_2_CO_3_) at low pressures

**DOI:** 10.1039/d6ra06420e

**Published:** 2026-09-25

**Authors:** Dominik Spahr, Lukas Brüning, Lars Ehm, Lkhamsuren Bayarjargal, Maxim Bykov, Victor Milman, Vitali B. Prakapenka, Stella Chariton, Zhenxian Liu, Björn Winkler, Elena Bykova

**Affiliations:** a Goethe University Frankfurt, Institute of Geosciences Altenhöferallee 1 60438 Frankfurt Germany d.spahr@kristall.uni-frankfurt.de; b Stony Brook University, Department of Geosciences 255 Earth & Space Science Building Stony Brook NY 11794-2100 USA; c Goethe University Frankfurt, Institute of Inorganic and Analytical Chemistry Max-von-Laue-Straße 7 60438 Frankfurt Germany; d Dassault Systèmes BIOVIA, 22 Cambridge Science Park CB4 0FJ Cambridge UK; e University of Chicago, GeoSoilEnviroCARS Chicago IL 60637 USA; f University of Illinois Chicago, Department of Physics Chicago IL 60607–7059 USA

## Abstract

Carbonic acid (H_2_CO_3_) has been detected on the surfaces of icy moons and has been suggested to occur in the interior of ice giants. Hence, understanding structure–property relations of phases in the H–C–O system is of significant interest to astrophysics and solid-state chemistry. Here we show, that at low pressures (≈9 GPa) two H_2_CO_3_ polymorphs can occur simultaneously at different temperatures. The two H_2_CO_3_ phases were obtained in different areas of a sample chamber of a diamond anvil cell after laser-heating a H_2_O + CO_2_ mixture. The orthorhombic crystal structure of H_2_CO_3_-*Pnma* was refined from synchrotron single crystal X-ray diffraction, including the position of the hydrogen atoms. The structure had previously been predicted, but had not been confirmed experimentally. Our density functional theory-based calculations reproduce the experimental Raman spectrum, confirming that the structural model of H_2_CO_3_-*Pnma* is appropriate. From the spatial phase distribution in the sample chamber we infer that H_2_CO_3_-*Pnma* is the low temperature polymorph of H_2_CO_3_-*P*2_1_/*n*, implying that the *p*,*T*-diagram of carbonic acid is more complex at low pressures than previously assumed.

## Abstract 2

H_2_CO_3_-*Pnma* is a low temperature carbonic acid polymorph, which can occur at the same pressure (∼9 GPa) than the high-temperature phase H_2_CO_3_-*P*2_1_/*n*. The crystal structure of H_2_CO_3_-*Pnma* was refined from synchrotron single crystal diffraction data.

## Graphical abstract

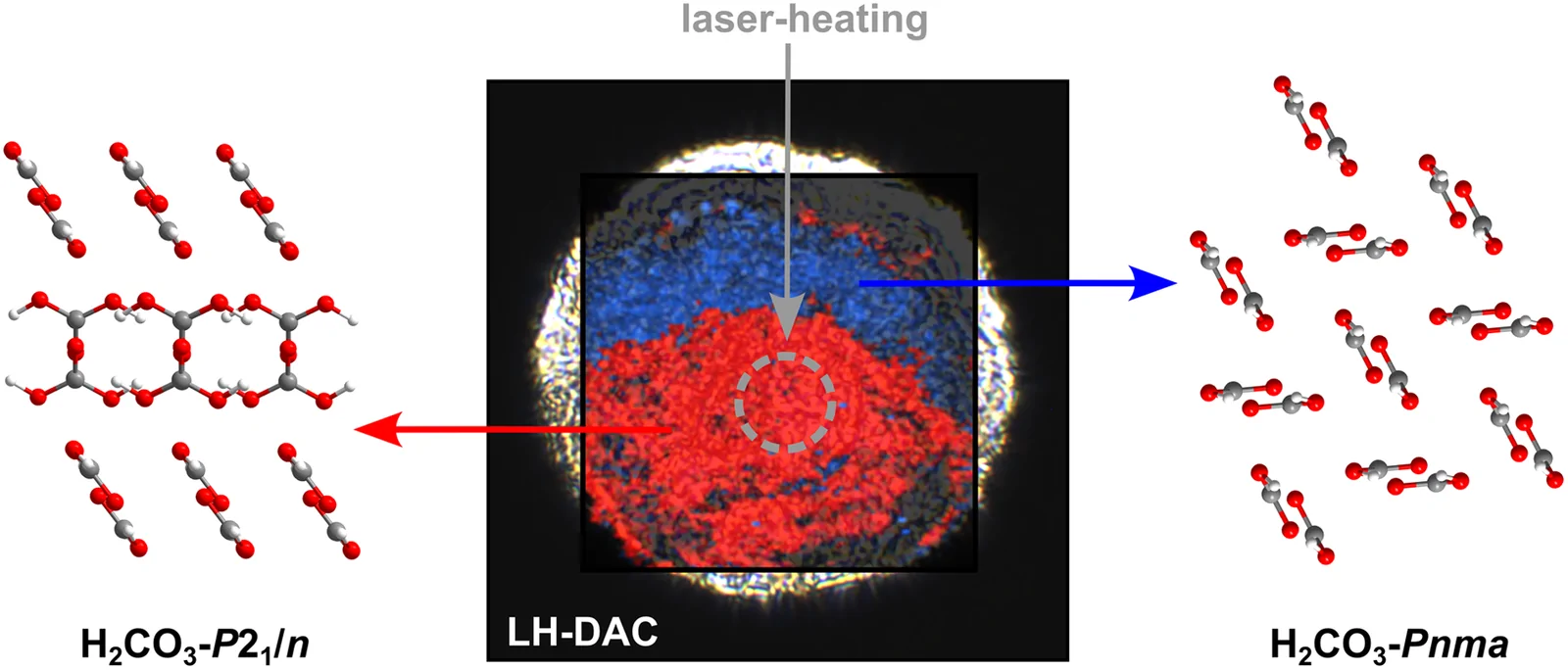

## Introduction

The H_2_O–CO_2_ system has gathered significant attention in the last decades due to its great relevance for astrophysical and planetary research. H_2_O as well as CO_2_ are major components of cometary nuclei and are abundant in the atmospheres and in the interiors of icy bodies throughout the solar system.^1–11^ In particular, carbonic acid (H_2_CO_3_) has been recently investigated in detail as it forms as a main product along the H_2_O–CO_2_ binary. Carbonic acid has been suggested as a reservoir of CO_2_ on the icy moons in our solar system.^3^ In addition, spectroscopic evidence for the presence of H_2_CO_3_ on the surface of Ganymede had been reported by the Jovian Infrared Auroral Mapper (JIRAM).^5,12^ Outside our solar system, the JWST detected H_2_O and CO_2_ in the atmosphere of the hot Super-Neptune WASP-166b and in potentially habitable water-rich planets such as LHS 1140 b and TOI-270 d.^13–15^

Recent experimental advances enabled the investigation of phase-relations in the H_2_O–CO_2_ system at elevated pressures.^16–24^ For example, a density functional theory (DFT) based crystal structure prediction study reported the existence of H_2_CO_3_-*Pnma* in the pressure range between 1 GPa and 44 GPa. It was proposed that the computed vibrational spectra were in agreement with the experimentally obtained IR- and Raman-spectra measured at ≈4 GPa in an earlier study.^16,17^ However, the reported identification in the IR- and Raman spectra remained ambiguous and no experimentally determined structure model for H_2_CO_3_-*Pnma* has been reported yet. In a combined neutron powder diffraction and DFT study on a deuterated sample at ≈2 GPa a monoclinic compound (H_2_CO_3_-*P*2_1_/*c*) was proposed, but as it is evident from the published data that the amount of H_2_CO_3_ in the sample was very small in comparison to the co-existing CO_2_-I phase (dry ice), and the refinements relied on constraints and restraints.^18^ At low temperatures and at 4 GPa and 6 GPa temperature-composition phase diagrams in the H–C–O system have been reported.^19^ However, the crystal structures of the investigated phases in their study remained undetermined due to the absence of high-quality diffraction data. In a follow-up study a single crystal structure determination of carbonic acid monohydrate (H_2_CO_3_·H_2_O-*P*1) was reported (at 6.5 GPa), providing the first robust experimental structural model of a carbonic acid phase at elevated pressures.^20^ Nevertheless, this study did not employ any DFT-based calculations or spectroscopic measurements to confirm the experimentally derived structural model. Recently, Berni *et al.*^21,22^ reported the existence of carbonic acid monohydrate at low pressures and low temperatures (<450 K). This would support the hypothesis that carbonic acid monohydrate could be a potential deep carbon reservoir in habitable water worlds.^25^ Berni *et al.*^21,22^ concluded that CO_2_ clathrate was a necessary prerequisite for the formation of carbonic acid hydrate. However, their conclusions are based on powder diffraction data and Raman spectroscopy, while theoretical investigations based on DFT were not employed.^21,22^ Due to its relevance for astrophysical research of icy extraterrestrial bodies and different carbonic acids phases were very recently investigated regarding double superionicity over a wide pressure range.^26^

In our recent study, we synthesized water-free carbonic acid from a mixture of H_2_O + CO_2_ in a laser-heated diamond anvil cell (LH-DAC) and determined the crystal structure (H_2_CO_3_-*P*2_1_/*n*) at a pressure of ≈8 GPa from X-ray single crystal diffraction data.^23^ At higher pressures (40(2) GPa) we obtained polymeric carbonic acid (H_2_CO_3_-*Cmc*2_1_) following the same experimental approach.^24^ The structural model derived in that study is in agreement with the results of an earlier crystal structure prediction study in the H–C–O system.^16^ From our previous DFT-based calculations in the athermal limit we found that the three structural models of H_2_CO_3_ reported for pressures ≤10 GPa (*P*2_1_/*n*, *Pnma* and *P*2_1_/*c*) all have enthalpies within a few kJ mol^−1^.^16,18,23^ Hence, it is possible that moderate changes in the *p*,*T*-conditions may lead to the formation of different polymorphs *i.e.* H_2_CO_3_-*Pnma* or H_2_CO_3_-*P*2_1_/*c*. This is supported by the observation that the energy difference for *cis*–*cis* and *cis*–*trans* conformations in H_2_CO_3_ is small (≈1.6 kJ mol^−1^).^27^ Here we investigated the reaction of H_2_O with CO_2_ in a similar manner than for the synthesis of H_2_CO_3_-*P*2_1_/*n* at 8(1) GPa,^23^ however, we created a steep thermal gradient in the sample chamber by focusing the heating spot of the CO_2_-laser on the lower half of the sample chamber. This allows us to investigate if different H_2_CO_3_ polymorphs will form in the colder part of the sample chamber, away from the heated spot. Based on synchrotron single crystal X-ray diffraction, Raman spectroscopy, synchrotron IR-measurements and DFT-based calculations we show that H_2_CO_3_-*Pnma* is the low-temperature carbonic acid polymorph at low pressures. A phase formed in the H–C–O system at lower temperatures than H_2_CO_3_-*P*2_1_/*n*, is of significant interest for the research of the icy moons in our solar system and needs to be taken into account for modeling their interiors.

## Results and discussion

The experiments at elevated pressures were carried out in LH-DACs and the H_2_O + CO_2_ mixture was prepared by cryogenic loading in a similar manner to that of the synthesis of H_2_CO_3_-*P*2_1_/*n* and H_2_CO_3_-*Cmc*2_1_.^23,24^ First, the DAC was cooled down in air and we monitored the precipitation of H_2_O in the sample chamber. After a sufficient amount of H_2_O-ice was condensed into the sample chamber, we switched on the CO_2_ gas jet and CO_2_-I (dry ice) was directly condensed into the sample chamber. After the sample chamber was completely covered with dry-ice, the DAC was tightly closed and compressed to the target pressure without intermediate heating (Fig. 1a). At the target pressure of the experiment (≈9 GPa) we employed Raman spectroscopy to confirm the presence of CO_2_ and H_2_O in the sample chamber. At pressures ≤12 GPa, CO_2_-I (*Pa*3̄) is the stable CO_2_-phase up to its melting temperature and we could identify the characteristic Raman modes of CO_2_-I at low wavenumbers (<300 cm^−1^).^28–30^ In addition, our experimental Raman spectra also show the characteristic Raman modes of H_2_O-VII (*Pn*3̄*m*) at high wavenumbers (≈3000–3400 cm^−1^) at the target pressure.^31,32^

**Fig. 1:**
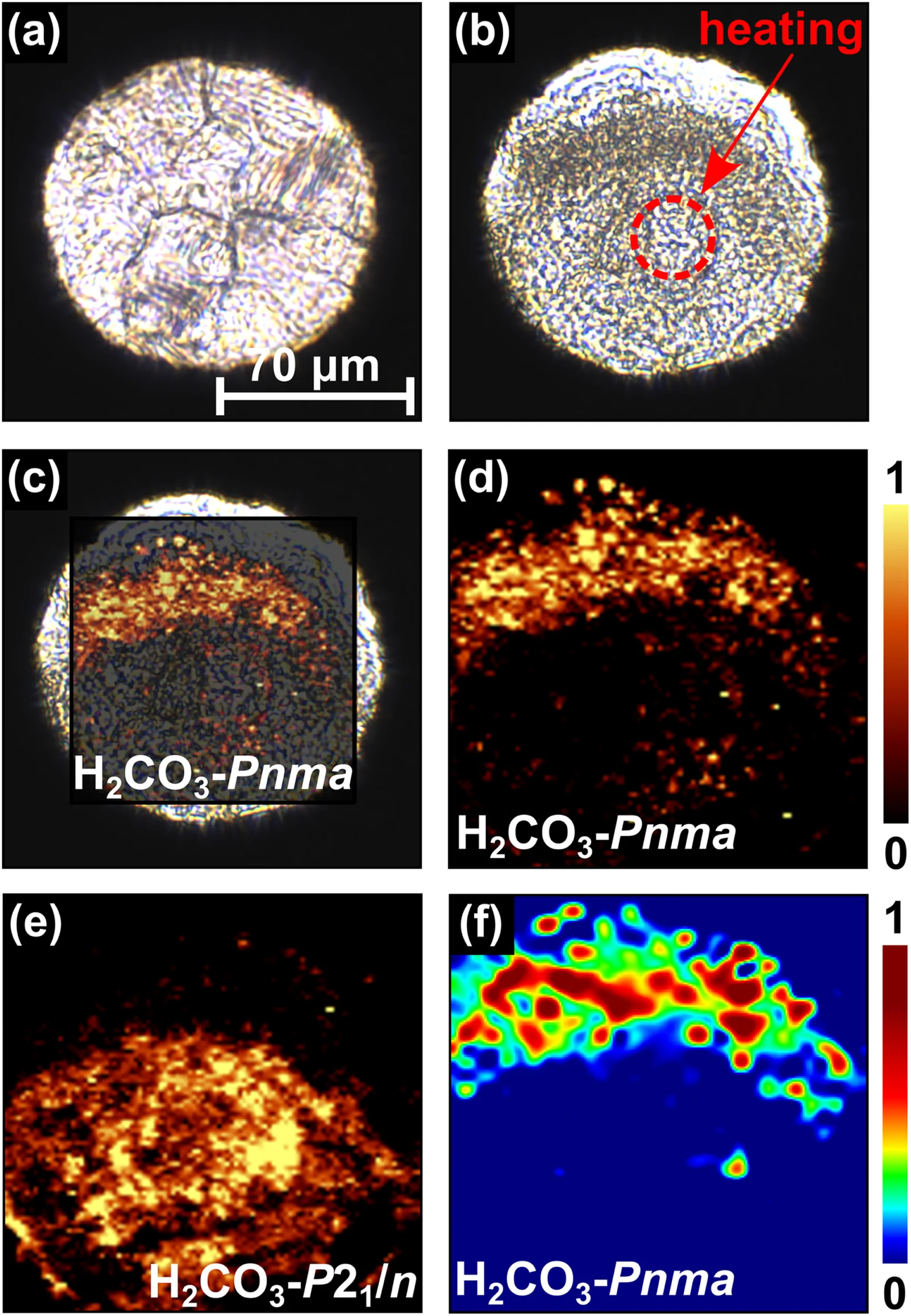
(a) Sample chamber of the DAC before laser-heating the H_2_O + CO_2_ mixture at 9(1) GPa. (b) Sample chamber of the DAC after laser-heating. (c) 2D-Raman map showing the distribution of H_2_CO_3_-*Pnma* overlayed on a picture of the sample chamber. (d) Raman map of H_2_CO_3_-*Pnma* (≈618 cm^−1^). (e) Raman map of H_2_CO_3_-*P*2_1_/*n* (≈681 cm^−1^). (f) XRD-map of H_2_CO_3_-*Pnma*, where the intensity of the (111) reflection at 2*θ* ≈ 6.03° is plotted.

After reaching ≈9 GPa, the H_2_O + CO_2_ mixture was laser-heated up to the beginning of optically detectable thermal radiation, which typically becomes visible at *T* ≈ 800 K.^33^ The CO_2_-laser was focused to the lower half of the sample chamber (Fig. 1b) in order to retain a “cold” region in the upper part of the sample chamber. The heating time was 45 minutes. During heating, no phase transition is expected in CO_2_-I and H_2_O-VII.^30,34^ The experimental Raman spectrum of CO_2_-I after the heating is in good agreement with the one from our DFT-based calculations (Fig. 2a). Employing spatially resolved Raman spectroscopy on a grid across the sample chamber we found that H_2_CO_3_-*P*2_1_/*n* is present in the heated area (Fig. 1e), in agreement with our previous experiments.^23^ The Raman spectrum of H_2_CO_3_-*P*2_1_/*n* is dominated by a strong Raman mode at ≈1090 cm^−1^ and three characteristic Raman modes between 550 cm^−1^ and 700 cm^−1^ (Fig. 2b and d). It is in good agreement with our DFT-calculations. In addition, we found that a second phase with a strong Raman mode at ≈1090 cm^−1^ is present in the sample chamber (Fig. 2c). Raman modes occurring at this wavenumber are characteristic for C–O stretching vibrations in compounds containing [CO_3_]^2−^-groups as structural building blocks such as carbonic acid (*e.g.* H_2_CO_3_-*P*2_1_/*n*) or sp^2^-carbonates (*e.g.* CaCO_3_).^23,35^ However, the unknown phase shows noticeable differences in the Raman modes between 550 cm^−1^ and 700 cm^−1^ in comparison with H_2_CO_3_-*P*2_1_/*n* (Fig. 2d and e). From the 2D Raman mapping of the sample chamber we found that the unknown [CO_3_]^2−^-group containing phase is only present in a rim around the laser-heated area (Fig. 1d), where H_2_CO_3_-*P*2_1_/*n* is present. Based on the spatial phase distribution and the focus of the laser beam to the heated area (where H_2_CO_3_-*P*2_1_/*n* is present) we infer that the unknown, [CO_3_]^2−^-group containing phase, is a “low-temperature” phase. However, it cannot be excluded that kinetic effects also contribute to the formation of different phases in the sample chamber. It is well established that laser-heating in DACs always suffers from large temperature gradients and the actual temperature is strongly dependent on the coupling of the laser with the sample, especially at lower temperatures.^36^ Hence, we assume that also in the heated area (Fig. 1d) a noticeable temperature gradient is present.

**Fig. 2:**
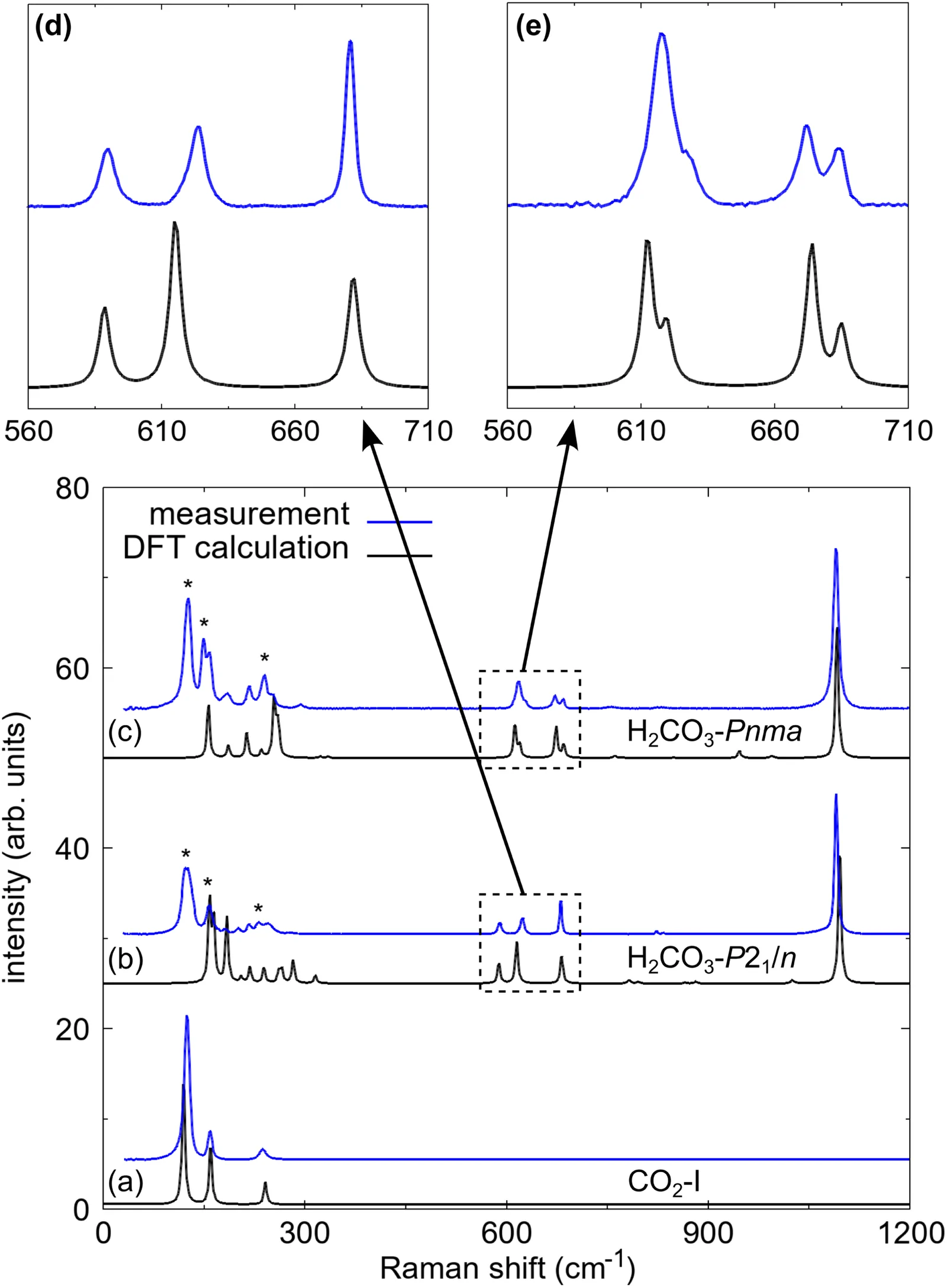
Raman spectroscopy at 9(1) GPa after laser heating the H_2_O + CO_2_ mixture: (a) Raman spectra for CO_2_-I. (b) Raman spectra for H_2_CO_3_-*P*2_1_/*n*. (c) Raman spectra for H_2_CO_3_-*Pnma*. Enlargement of the region between 560 cm^−1^ and 710 cm^−1^: (d) for H_2_CO_3_-*P*2_1_/*n* and (e) for H_2_CO_3_-*Pnma*. Experimental Raman spectra are shown in blue, DFT-based calculations are shown in black. The Raman shifts of the theoretical spectra were scaled by 1–2%. Peaks of CO_2_-I in the Raman spectrum of the two H_2_CO_3_ polymorphs are marked by an asterisk (*).

After the Raman mapping, we performed synchrotron X-ray diffraction in order to determine the crystal structure of the yet unknown phase. We employed the recently upgraded beam line 13-ID-D (GSECARS) at the Advanced Photon Source (APS) using a ≈1.5 × 1.5 µm^2^-sized X-ray beam. In the first step, we collected X-ray powder diffraction data on a 2D-grid across the sample chamber. The XRD-map of a reflection at 2*θ* ≈ 6.03° (Fig. 1f), is in very good agreement with the Raman map of the unknown phase (Fig. 1d) and the reflection cannot be assigned to H_2_CO_3_-*P*2_1_/*n*, CO_2_-I or H_2_O-VII.^23,37,38^ Hence, we collected diffraction data suitable for single crystal X-ray diffraction on grid positions in this area of the sample chamber. From the single crystal X-ray diffraction data collected at 9(1) GPa we solved the crystal structure of the unknown phase. The new phase has H_2_CO_3_ composition and crystallizes in the orthorhombic space group *Pnma* with *Z* = 4 (Fig. 3a). The lattice parameters at 9(1) GPa are *a* = 4.994(5) Å, *b* = 6.547(2) Å and *c* = 5.330(2) Å (*V* = 174.2(2) Å^3^). It is distinct from H_2_CO_3_-*P*2_1_/*n* (Fig. 3b), which is also present in the sample chamber.^23^ The low *R*_1_-value of 4.4% in combination with a high reflection-to-parameter-ratio (12.5 : 1) are indicative of a very good structure refinement (see Table S1 in the SI). The displacement parameters of the carbon and oxygen atoms were refined anisotropically and no constraints or restraints had to be introduced for the structure refinement. The experimentally determined crystal structure of H_2_CO_3_-*Pnma* is in agreement with our DFT-based full geometry optimization for the same pressure (see Table S1 in the SI). This structural model for carbonic acid has been predicted earlier by evolutionary algorithm in the H–C–O ternary system, with a suggested stability field between 1 GPa and 44 GPa.^16^ For 1 GPa lattice parameters of *a* = 5.2199 Å, *b* = 6.5624 Å and *c* = 5.6268 Å (*V* = 192.75 Å^3^) were reported based on their calculations. Employing our DFT-based calculations at 1 GPa, using the same starting model of H_2_CO_3_-*Pnma*, resulted in the same structure, but with noticeably different *a*- and *c*-lattice parameters (*a* = 5.5263 Å, *b* = 6.5201 Å, *c* = 6.1642 Å and *V* = 222.11 Å^3^). The calculations differ in several respects (PAW *vs.* plane wave/pseudopotentials, *k*-point sampling, different approach to the dispersion correction), but while the lattice parameters differ, the geometries of the H_2_CO_3_-molecules, their orientation and their packing obtained from the two approaches are very similar.

**Fig. 3:**
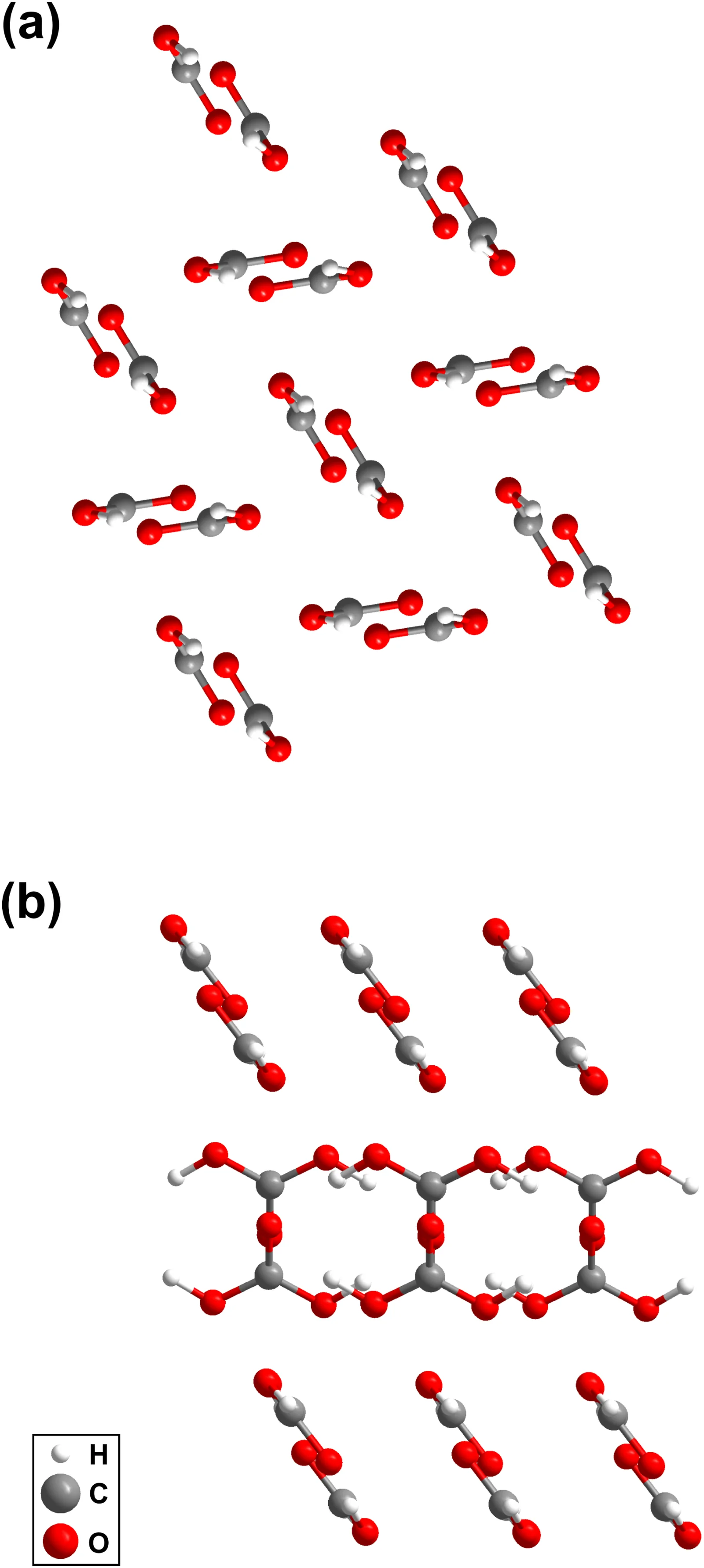
Comparison of the arrangement of the H_2_CO_3_ molecules in (a) H_2_CO_3_-*Pnma* (9(1) GPa) and (b) H_2_CO_3_-*P*2_1_/*n* (8(1) GPa).^23^ Structural models of both carbonic acid polymorphs were obtained experimentally by synchrotron single-crystal X-ray diffraction in LH-DACs.

We employed DFPT-calculations to derive a theoretical Raman spectrum for H_2_CO_3_-*Pnma*. The experimental Raman spectrum is accurately reproduced by our calculations (Fig. 2c), confirming that the structural model of H_2_CO_3_-*Pnma* is appropriate and that our DFT-based calculations accurately describe our experimental data. In particular, the Raman modes between 550 cm^−1^ and 700 cm^−1^ can be used to distinguish between the different H_2_CO_3_ polymorphs at this pressure (Fig. 2d and e). While our experimental and theoretical Raman spectra for H_2_CO_3_-*P*2_1_/*n* clearly show three separate peaks (Fig. 2d), in H_2_CO_3_-*Pnma* one strong peak with a tiny shoulder and two peaks very close to each other are present (Fig. 2e). In contrast, the [CO_3_]^2−^-stretching mode at ≈1090 cm^−1^ is identical for both H_2_CO_3_ polymorphs present in the sample chamber of the DAC (Fig. 2b and c). Due to an overlap with the Raman modes of CO_2_-I at low wavenumbers (<300 cm^−1^) an unambiguous assignment of the lattice modes is challenging.

Similar to H_2_CO_3_-*P*2_1_/*n*^23^ the crystal structure of H_2_CO_3_-*Pnma* is characterized by H_2_CO_3_ molecules containing nearly trigonal-planar [CO_3_]^2−^-groups. The H_2_CO_3_ molecules are connected to each other by two hydrogen bonds. The experimental O⋯H distance (1.69(3) Å) is in agreement with the values observed for H_2_CO_3_-*P*2_1_/*n* at 8(1) GPa (1.71(6) Å/1.63(5) Å)^23^ and slightly longer than the value derived from our DFT-based calculations for H_2_CO_3_-*Pnma* (1.46 Å). The position of the hydrogen atom in H_2_CO_3_-*Pnma* could directly be located in the difference Fourier map during the crystal structure refinement when the hydrogen atoms were omitted (Fig. 4a). After introducing the hydrogen atom in the structure, the residual electron density at the positions vanishes (Fig. 4b). The refinement with the hydrogen atom in place resulted in a decrease of the *R*_1_-value by ≈1.5%. The successful refinement of hydrogen atom positions from synchrotron single-crystal X-ray diffraction collected on a multi-grain sample has been enabled by the recent technical developments in synchrotron X-ray diffraction. The latest generation of synchrotron sources (*e.g.* APS-U or ESRF-EBS) can provide a strongly focused X-ray beam in combination with a high photon flux on the sample. State-of-the-art single-photon-counting detectors with small pixel sizes (*e.g.* Eiger2 X) are highly sensitive, allow long exposure times without oversaturation and offer a very high signal-to-noise ratio. In combination with highly precise and vibration-free sample stages this enables single-crystal X-ray diffraction experiments on very challenging samples such as carbonic acid. We have exploited this technical progress succesfully earlier with multi-grain single crystal studies of H_2_CO_3_-*P*2_1_/*n*, H_2_CO_3_-*Cmc*2_1_, Li[HC_2_O_5_] or B[µ-H(CO_3_)_2_] at elevated pressures in DACs, where we demonstrated that in favorable circumstances, *i.e.* in the absence of strong scatterers, we can localize the hydrogen positions reliably with our experimental approach.^23,24,39,40^

**Fig. 4:**
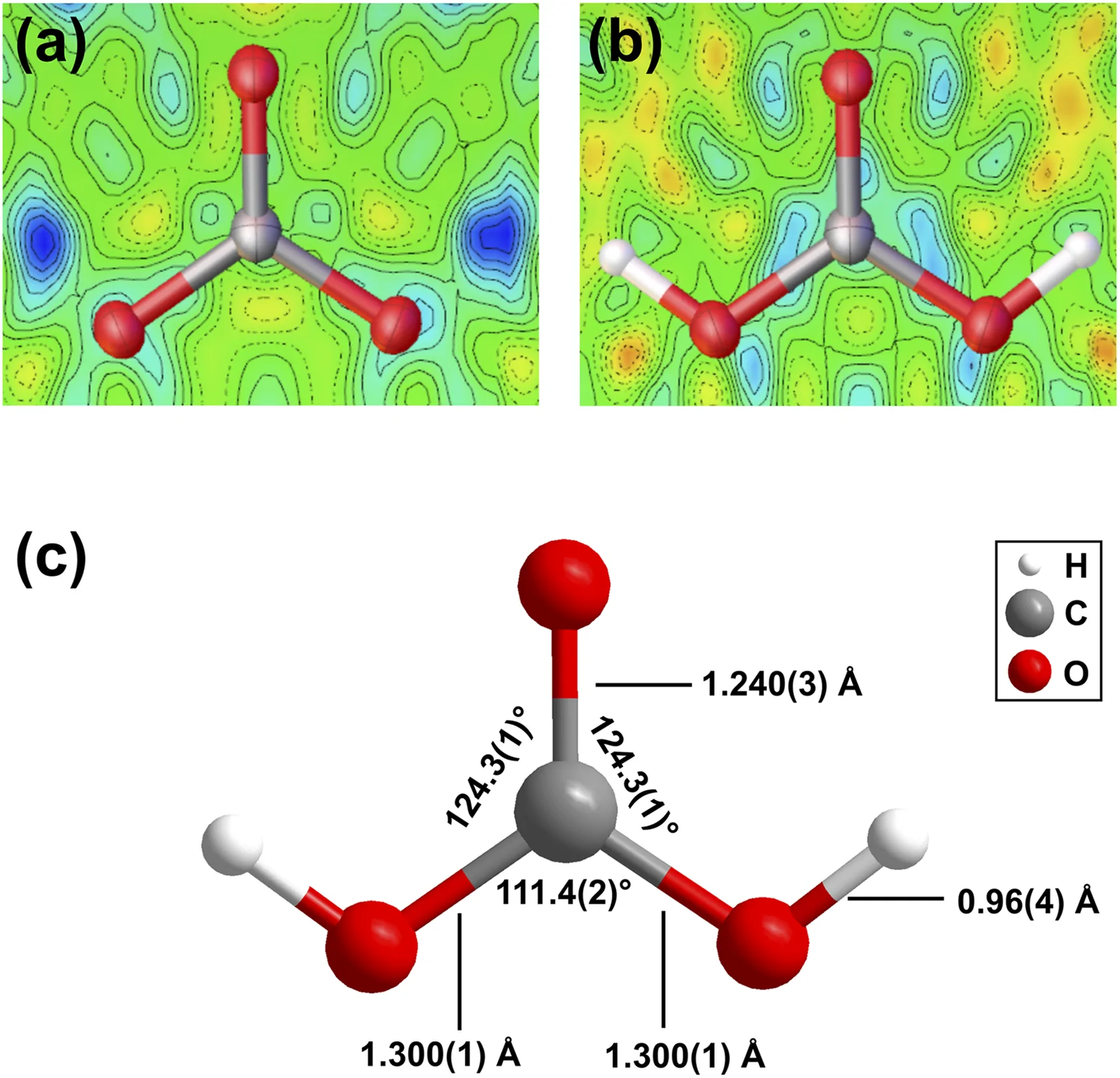
Difference Fourier map around the H_2_CO_3_ molecule in H_2_CO_3_-*Pnma* at 9(1) GPa: (a) refinement without the hydrogen atom and (b) with the hydrogen atom connected to the oxygen atom. (c) Geometry of the H_2_CO_3_ molecule from single crystal structure refinement.

The geometry of the H_2_CO_3_ molecule in H_2_CO_3_-*Pnma* (Fig. 4c) is in good agreement with our results for H_2_CO_3_-*P*2_1_/*n* at similar pressures. In H_2_CO_3_-*Pnma* we observed two different C–O bond lengths (1.240(3) Å and 1.300(1) Å) similar to H_2_CO_3_-*P*2_1_/*n* (1.267(3) Å and 1.297(3) Å/1.306(3) Å).^23^ In addition, in both polymorphs the C–O bond to the non-protonated oxygen atom is significantly shorter than the other two C–O bonds, where the oxygen atom is connected to a hydrogen atom. Our results for H_2_CO_3_-*Pnma* are reproduced by our DFT calculations and are in agreement with a structural model predicted earlier.^16^ In our DFT-calculations the shorter C–O bond (1.261 Å) has a Mulliken bond population of 0.91 e^−^ Å^−3^, while the longer ones (1.285 Å) have a bond population of 0.79 e^−^ Å^−3^. These values are in agreement with the bond populations in H_2_CO_3_-*P*2_1_/*n* (0.95 e^−^ Å^−3^ and 0.76 e^−^ Å^−3^).^23^

Experimentally, we observed a O–H bond distance of 0.96(4) Å, which is noticeably shorter than the value derived from our DFT-calculations (1.03 Å). However, within the experimental uncertainties this confirms the high-quality of our experimental data as it is generally accepted that DFT model calculations can reliably predict hydrogen positions.^41^ The covalent O–H bond has a Mulliken bond population of 0.49 e^−^ Å^−3^, while the O⋯H bond has a population of 0.19 e^−^ Å^−3^. These values are in agreement with our DFT-results for H_2_CO_3_-*P*2_1_/*n* (0.51 e^−^ Å^−3^ and 0.15 e^−^ Å^−3^) obtained earlier.^23^ In addition, our DFT-calculations show that the C–O–H angle in H_2_CO_3_-*Pnma* (112.9°) is almost identical to the ones in H_2_CO_3_-*P*2_1_/*n* (110.2° and 110.5°). In H_2_CO_3_-*Pnma* we observed two different O–C–O angles (111.4(2)° and 124.3(1)°) experimentally, where the smaller angle is between the two oxygen atoms connected to the hydrogen atoms. This geometry is similar to the ones obtained experimentally in H_2_CO_3_-*P*2_1_/*n* where the corresponding values are 113.2(2)°, 123.1(2)° and 123.8(3)°.^23^ Moreover, it demonstrated the high-quality of our experimental X-ray diffraction data as our DFT-calculations for H_2_CO_3_-*Pnma* resulted in angles of 113.6° and 123.2°. The geometry of the H_2_CO_3_ molecules in H_2_CO_3_-*Pnma* as well as in H_2_CO_3_-*P*2_1_/*n* in is *cis*–*cis* conformation.^23^ This is in contrast to their geometry with *cis*–*trans* conformation in H_2_CO_3_·H_2_O-*P*1 observed at similar pressures.^20^

While the geometry of the H_2_CO_3_ molecules in H_2_CO_3_-*Pnma* and H_2_CO_3_-*P*2_1_/*n*, in *cis*–*cis* confirmation, is almost identical, their crystal structures are noticeably different (Fig. 3). This difference results solely from a different packing of the H_2_CO_3_ molecules. The structure of H_2_CO_3_-*P*2_1_/*n* can be described by an alternating stacking of two layers with different orientated H_2_CO_3_ molecules (Fig. 3b).^23^ In contrast, in H_2_CO_3_-*Pnma* the two layers of H_2_CO_3_ molecules are only rotated by 90° with respect to each other and can be matched by a simple rotation (Fig. 3a). This results in a “zigzag” pattern of the H_2_CO_3_ layers. Within the layers the H_2_CO_3_ molecules are connected by hydrogen bonding with an almost identical geometry in both polymorphs.

In addition to Raman spectroscopy, we employed synchrotron based IR spectroscopy on the LH-DAC containing H_2_CO_3_-*Pnma* and H_2_CO_3_-*P*2_1_/*n*, in order to assess the IR-data obtained at ≈4 GPa in an earlier study.^17^ In our IR-data collected at 9(1) GPa we could clearly observe the characteristic peaks corresponding to CO_2_ and to H_2_O,^42,43^ without the presence of peaks corresponding to H_2_CO_3_, in a small region of the sample chamber where no reaction had occurred (Fig. S2, in the SI). In the laser heated area we observed the characteristic IR-signature of H_2_CO_3_ in a region between 1200 cm^−1^ and 2000 cm^−1^ (Fig. S2, in the SI), in agreement with our DFT-based calculations for both phases. The peaks observed in our measurements are in agreement with the IR-spectra obtained earlier at ≈4 GPa.^17^ They also agree with the IR-spectra obtained for H_2_CO_3_ at ambient pressure and low temperatures.^3^ Unfortunately, the difference between H_2_CO_3_-*Pnma* and H_2_CO_3_-*P*2_1_/*n* could not be resolved in the IR-spectra. In addition, the strongest IR-peak of both phases, according to our DFT-calculations, is present in the region between 2200 cm^−1^ and 2400 cm^−1^ and overlaps with the strongest peak of CO_2_ (Fig. S2, in the SI). In our experimental data the H_2_CO_3_ peak it is only present as a small shoulder of the CO_2_-peak. Our results clearly show the limitations of IR-spectroscopy for the study of these phases and that H_2_CO_3_-*Pnma* and H_2_CO_3_-*P*2_1_/*n* could not be distinguished. However, in combination with our DFT-calculations, the IR-spectra of H_2_CO_3_ can be employed to identify the presence of solid H_2_CO_3_ by remote sensing in meteorites or on icy moons or planets.^1–5^

Previously we already demonstrated that the phonon density of states (PDOS) of H_2_CO_3_-*Pnma* as well as of H_2_CO_3_-*P*2_1_/*n* show no negative frequencies and that the phonon dispersion curves indicate that both phases are dynamically stable at 8 GPa.^23^ This is in agreement with our experimental observation of both phases here at a similar pressure (≈9 GPa). In addition, here we employed our DFT-calculations in order to understand the *p*,*V*-relation for H_2_CO_3_-*Pnma* in comparison to H_2_CO_3_-*P*2_1_/*n*. We fitted the theoretical *p*,*V*-data with an EoS-fit in order to determine the bulk modulus (Fig. S3, in the SI). We obtained a bulk modulus of *K*_0_ = 12.3(3) GPa with *K*_p_ = 5.8(1) for H_2_CO_3_-*Pnma*. Within the expected uncertainties these results are identical with the values derived for H_2_CO_3_-*P*2_1_/*n* (*K*_0_ = 14.2(4) GPa with *K*_p_ = 6.1(1)).^23^ In addition the refined ambient pressure volume *V*_0_ is similar for H_2_CO_3_-*Pnma* (238.3(6) Å ^3^) and H_2_CO_3_-*P*2_1_/*n* (231.5(6) Å ^3^). The similar bulk moduli and volumes of both phases are not surprising as their different crystal structures only result from a different packing of the same H_2_CO_3_ molecules.

## Conclusion

In summary, our experimental data in the H–C–O system demonstrate that the *p*,*T*-diagram of H_2_CO_3_ at low pressures is more complex than was assumed previously. Based on the spatial phase distribution in the sample chamber we conclude that H_2_CO_3_-*Pnma* is a low temperature carbonic acid polymorph, which can be formed at the same pressure as the high-temperature polymorph H_2_CO_3_-*P*2_1_/*n*.^23^ The crystal structure of H_2_CO_3_-*Pnma* was refined from synchrotron single crystal X-ray diffraction data at 9(1) GPa, including the positions of the hydrogen atoms and confirms an earlier predicted structural model.^16^ H_2_CO_3_-*P*2_1_/*c*, described in a combined neutron powder diffraction and DFT study at ≈2 GPa was not observed here.^18^ Our diffraction data show that the geometry H_2_CO_3_ molecules is almost identical in H_2_CO_3_-*Pnma* and H_2_CO_3_-*P*2_1_/*n* and that the molecules are connected by hydrogen bonding within layers. In both phases the H_2_CO_3_ molecules occur in *cis–cis* confirmation. The difference in their crystal structure results only from a different arrangement of these H_2_CO_3_-layers.

Our DFT calculations reproduce the experimental Raman spectrum for H_2_CO_3_-*Pnma* satisfactorily. Moreover, we show that H_2_CO_3_-*Pnma* and H_2_CO_3_-*P*2_1_/*n* can be distinguished using Raman spectroscopy in the frequency range between 550 cm^−1^ and 700 cm^−1^, which will facilitate their identification in future experiments. Our synchrotron IR-measurements show a characteristic signature for H_2_CO_3_, in agreement with earlier studies.^3,17^ However, a differentiation between H_2_CO_3_-*Pnma* and H_2_CO_3_-*P*2_1_/*n* is not possible based on IR-data. Nevertheless IR spectroscopy will allow their identification by remote sensing of icy bodies in our solar system.^1–5^ Our DFT-calculations show that H_2_CO_3_-*Pnma* and H_2_CO_3_-*P*2_1_/*n* have a very similar bulk modulus. In addition, the calculations show that H_2_CO_3_-*Pnma* has a slightly higher density than H_2_CO_3_-*P*2_1_/*n* at the same pressure (0.2% at 10 GPa and 0.6% at 20 GPa). However, a phase transition between both phases would most probably not result in phase segregation inside of ice bodies, but it is possible, that both phases can occur in their interiors, depending on the temperature.

Due to the abundance of H_2_O and CO_2_ in cometary nuclei, in atmospheres and in the interiors of icy bodies throughout the solar system phases in the H–C–O system had always been of great interest for astrophysical research.^1–11^ However, only a very limited number of experimental crystal structure determinations in this ternary system have been reported up to now, which are, in turn, required to calculate physical properties of these phases for model calculations of icy bodies or to derive vibrational spectra required for remote sensing. Our experimental results in the H–C–O system demonstrate that the *p*, T-diagram of H_2_CO_3_ at low pressures is more complex than was assumed previously assumed and that *e.g.* IR-spectra are insufficient for a phase identification. Reliable structural models in the H–C–O system are also required for further theoretical investigations such as those by Deng *et al.* (2026)^26^ who studied double superionicity in the system H–C–O and suggested that different carbonic acids phases *trans*-form into a hydrogen-diffusive superionic phase and subsequently become C–H doubly superionic. We are confident that our study, which combines single crystal X-ray diffraction, spectroscopic measurements and DFT-based calculations provides a road map to avoid misidentification of phases due to problematic interpretations of powder diffraction data.

## Author contributions

D. S., L. Br., L. E., L. Ba. and Z. L. performed experiments. V. M. and B. W. performed DFT calculations. V. B. P., S. C. and Z. L. managed the synchrotron beam lines. B. W., E. B. and M. B. supervised the project.

## Conflicts of interest

There are no conflicts to declare.

## Supplementary Material

RA-016-D6RA06420E-s001

## Data Availability

CCDC 2566151 contains the supplementary crystallographic data for this paper.^44^ All study data are included in the article and/or in the supplementary information (SI). Supplementary information: experimental and computational details, crystallographic data, X-ray single crystal measurements, IR-spectroscopy and DFT-based calculations. See DOI: https://doi.org/10.1039/d6ra06420e.

## References

[cit1] Strazzulla G., Brucato J. R., Cimino G., Palumbo M. E. (1996). Planet. Space Sci..

[cit2] Hage W., Liedl K. R., Hallbrucker A., Mayer E. (1998). Science.

[cit3] Jones B. M., Kaiser R. I., Strazzulla G. (2014). ApJ.

[cit4] Sanz-Novo M., Rivilla V. M., Jiménez-Serra I., Martín-Pintado J., Colzi1 L., Zeng S., Megías A., López-Gallifa A., Martínez-Henares A., Massalkhi S., Tercero B., de Vicente P., Martín S., San Andrés D., Requena-Torres M. A. (2023). ApJ.

[cit5] Tosi F., Mura A., Cofano A., Zambon F., Glein C. R., Ciarniello M., Lunine J. I., Piccioni G., Plainaki C., Sordini R., Adriani A., Bolton S. J., Hansen C. J., Nordheim T. A., Moirano A., Agostini L., Altieri F., Brooks S. M., Cicchetti A., Dinelli B. M., Grassi D., Migliorini A., Moriconi M. L., Noschese R., Scarica P., Sindoni G., Stefani S., Turrini D. (2024). Nat. Astron..

[cit6] Crovisier J. (2006). Faraday Discuss..

[cit7] Crovisier J. (2006). Mol. Phys..

[cit8] Schiltz L., Escribano B., Muñoz Caro G. M., Cazaux S., del Burgo Olivares C., Carrascosa H., Boszhuizen I., González Díaz C., Chen Y.-J., Giuliano B. M., Caselli P. (2024). A&A.

[cit9] Trumbo S. K., Brown M. E. (2023). Science.

[cit10] Soderlund K. M., Rovira-Navarro M., Le Bars M., Schmidt B. E., Gerkema T. (2024). Annu. Rev. Mar. Sci..

[cit11] Journaux B., Kalousová K., Sotin G., Tobie C., Vance S., Saur J., Bollengier O., Noack L., Rückriemen-Bez T., Van Hoolst T., Soderlund K. M., Brown J. M. (2020). Space Sci. Rev..

[cit12] Protopapa S., Raut U., Wong I., Stansberry J., Villanueva G. L., Cook J., Holler B., Grundy W. M., Brunetto R., Cartwright R. J., Mamo B., Emery J. P., Parker A. H., Guilbert-Lepoutre A., Pinilla-Alonso N., Milam S. N., Hammel H. B. (2024). Nat. Commun..

[cit13] Mayo A. W., Fortenbach C. D., Louie D. R., Dressing C. D., Turtelboom E. V., Giacalone S., Harada C. K. (2025). AJ.

[cit14] Damiano M., Bello-Arufe A., Yang J., Hu R. (2024). ApJL.

[cit15] Benneke B. , Roy P.-A., Coulombe L.-P., Radica M., Piaulet C., Ahrer E.-M., Pierrehumbert R., Krissansen-Totton J., Schlichting H. E., Hu R., Yang J., Christie D., Thorngren D., Young E. D., Pelletier S., Knutson H. A., Miguel Y., Evans-Soma T. M., Dorn C., Gagnebin A., Fortney J. J., Komacek T., MacDonald R., Raul E., Cloutier R., Acuna L., Lafreniére D., Cadieux C., Doyon R., Welbanks L. and Allart R., arXiv, 2024, preprint, arXiv:2403.03325, 10.48550/arXiv.2403.03325

[cit16] Saleh G., Oganov A. R. (2016). Sci. Rep..

[cit17] Wang H., Zeuschner J., Eremets M., Troyan I., Willams J. (2016). Sci. Rep..

[cit18] Benz S., Chen D., Möller A., Hofmann M., Schnieders D., Dronskowski R. (2022). Inorganics.

[cit19] Abramson E. H., Bollengier O., Brown J. M. (2017). Sci. Rep..

[cit20] Abramson E. H., Bollengier O., Brown J. M., Journaux B., Kaminsky W., Pakhomova A. (2018). Am. Mineral..

[cit21] Berni S., Scelta D., Romi S., Fanetti S., Alabarse F., Pagliai M., Bini R. (2024). Angew. Chem., Int. Ed..

[cit22] Berni S., Scelta D., Romi S., Fanetti S., Alabarse F., Wehinger B., Bini R. (2025). Chem. Sci..

[cit23] Spahr E., Bykova D., Bayarjargal L., Bykov M., Brüning L., Kovalev V., Milman V., Giordano N., Liermann H.-P., Winkler B. (2025). Chem. Eur. J..

[cit24] Spahr D., Bayarjargal L., Brüning L., Kovalev V., Wedek L., Bykov M., Milman V., Giordano N., Winkler B., Bykova E. (2025). Commun. Chem..

[cit25] Marounina N., Rogers L. A. (2020). ApJ.

[cit26] Deng J., Gou H., Hu Q. (2026). Sci. Adv..

[cit27] Reisenauer H. P., Wagner J. P., Schreiner P. R. (2014). Angew. Chem., Int. Ed..

[cit28] Aoki K., Yamawaki H., Sakashita M., Gotoh Y., Takemura K. (1994). Science.

[cit29] Olijnyk H., Jephcoat A. P. (1998). Phys. Rev. B: Condens. Matter Mater. Phys..

[cit30] Scelta D., Dziubek K. F., Ende M., Miletich R., Mezouar M., Garbarino G., Bini R. (2021). Phys. Rev. Lett..

[cit31] Pruzan P., Chervin J. C., Gauthier M. (1990). Europhys. Lett..

[cit32] Hsieh W.-P., Chien Y.-H. (2015). Sci. Rep..

[cit33] Draper J. W., Edinb L. (1847). Dublin Philos. Mag. J. Sci..

[cit34] Hansen T. C. (2021). Nat. Commun..

[cit35] Bayarjargal L., Fruhner C.-J., Schrodt N., Winkler B. (2018). Phys. Earth Planet. Inter..

[cit36] Du Z., Amulele G., Benedetti L. R., Lee K. K. M. (2013). Rev. Sci. Instrum..

[cit37] Simon A., Peters K. (1980). Acta Crystallogr..

[cit38] Gajda R., Chodkiewicz M., Zhang D., Nguyen P., Prakapenka V., Wozniak K. (2025). IUCrJ.

[cit39] Spahr D., Bayarjargal L., Bykov M., Brüning L., Jurzick P. L., Milman V., Giordano N., Mezouar M., Winkler B. (2024). Angew. Chem., Int. Ed..

[cit40] Spahr D., Reuter T. H., Bykova E., Bayarjargal L., Brüning L., Kovalev V., Bykov M., Wedek L., Milman V., Jonathan W., Winkler B. (2025). Inorg. Chem..

[cit41] Milman V., Winkler B. (2001). Z. Kristallogr..

[cit42] Poteet C. A., Pontoppidan K. M., Megeath S. T., Watson D. M., Isokoski K., Bjorkman J. E., Sheehan P. D., Linnartz H. (2013). ApJ.

[cit43] Song M., Yamawaki H., Fujihisa H., Sakashita M., Aoki K. (2003). Phys. Rev. B: Condens. Matter Mater. Phys..

[cit44] CCDC 2566151: Experimental Crystal Structure Determination, 2026, 10.5517/ccdc.csd.cc2s492b

