# Supplementary material for: Polymorphism of carbonic acid (H2CO3) at low pressures
Source: RSC Adv. 2026 Sep 25;16(45):57695–701. doi: 10.1039/d6ra06420e (PMC13614443; doi:10.1039/d6ra06420e)
Supplement: Supplementary file 1 [file RA-016-D6RA06420E-s001.pdf]

Supplementary Information (SI) for RSC Advances.
This journal is © The Royal Society of Chemistry

Supplementary material: Polymorphism of carbonic acid (H_2CO_3) at low pressures

Dominik Spahr[*,a,b], Lukas Brüning[c], Lars Ehm[b], Lkhamsuren Bayarjargal[a], Maxim Bykov[c], Victor Milman[d], Vitali B. Prakapenka[e], Stella Chariton[e], Zhenxian Liu[f], Björn Winkler[a], Elena Bykova[a]

[a]*Goethe University Frankfurt, Institute of Geosciences, Altenhöferallee 1, 60438 Frankfurt, Germany*
[b]*Stony Brook University, Department of Geosciences, 255 Earth & Space Science Building, Stony Brook, NY 11794-2100, USA*
[c]*Goethe University Frankfurt, Institute of Inorganic and Analytical Chemistry, Max-von-Laue-Straße 7, 60438 Frankfurt, Germany*
[d]*Dassault Systèmes BIOVIA, 334 Cambridge Science Park, Cambridge CB4 0WN, United Kingdom*
[e]*University of Chicago, GeoSoilEnviroCARS, IL 60637, Chicago, USA*
[f]*University of Illinois Chicago, Department of Physics, IL 60607–7059, Chicago, USA*

1. Methods

1.1. High-pressure experiments

We used Boehler-Almax type DACs for our high-pressure experiments.[1] The DACs were equipped on both sides with diamonds having an opening angle of 70° in combination with a 350 µm sized culet. We employed Re-gaskets which we pre-indented to thicknesses of ≈ 50 µm for the experiments. We used a custom-built laser set-up to drill sample chambers with ≈ 120 µm into the pre-indented Re-gaskets to generate sample chambers. We added a ruby chip on the culet of the bottom diamond inside the sample chamber in order to determine the pressure during the experiment. We assume an error of at least 6% due to non-hydrostatic conditions in the sample chambers.[2]

For the cryogenic loading the DAC was placed on a liquid nitrogen cooled Cu-holder and cooled down using a custom-built cryogenic loading system (see Spahr *et al.* [3]). In the first step, the DAC was cooled down in air without any purge gas and we monitored the precipitation of H_2O in the sample chamber using an optical microscope and a camera. After a sufficient amount of H_2O-ice was condensed into the sample chamber, we switched on the CO_2 gas jet, which was directly directed at the gap between upper diamond and the gasket. Finally, the DAC was cooled down further to ≈ 120 K and CO_2-I (dry ice) was directly condensed into the gasket hole from the CO_2 gas jet. We used a small nozzle to align the CO_2 gas jet with 5 l min^{-1}. For the gas-jet during the cryogenic loading we used CO_2 gas as purchased (Nippon gases, purity ≥ 99.996%). After the sample chamber was completely covered with dry-ice, the DAC was tightly closed again and compressed to the target pressure without intermediate heating.

1.2. Laser heating

At the target pressures of the experiment the DACs with the H_2O + CO_2 mixture was laser-heated from one side in the lower half of the sample chamber. We used a custom-built set-up equipped with a Coherent Diamond K-250 pulsed CO_2 laser (λ = 10600 nm).[4] The laser power was adjusted to achieve a coupling of the laser to the sample using a laser power of ≤ 1 W. The sample was heated until the beginning of thermal radiation in the visible spectrum was visible, which is typically at $T \approx 800$ K.[5] We assume that the highest temperature achieved during the laser heating was $T_{max} \approx 800(200)$ K. The temperatures were determined by the two-color pyrometer method, employing Planck and Wien fits.[6] The heating time was ≈ 45 min. It is well established that laser-heating in DACs always suffers from large temperature gradients and the actual temperature is strongly dependent on the coupling of the laser with the sample, especially at lower temperatures. We estimate an uncertainty of at least ±20% of the nominal temperature in the laser-heated region depending on the focus of the laser beam, based on typical 2D temperature-gradient determination experiments performed in DACs.[7]

1.3. Raman spectroscopy

Raman spectroscopy was performed in the DAC using an Oxford Instruments WITec alpha 300R Raman imaging microscope. The Raman microscope was equipped with an Olympus SLMPan N 50× objective. The measurements were performed using the 532 nm laser. We employed the 1800 grooves mm^{-1} grating of the WITec UHTS 300S (VIS-NIR) spectrograph in combination with an Andor DR316B-LDC-DD CCD detector for the measurements. The applied laser power was 100 mW on the sample and the spot size of the Raman laser was ≈ 0.8 µm. We estimate a that the Raman laser penetrates ≈ 6 µm into the sample. Raman maps were measured on a grid with a step-size of 0.5 µm. The background of the Raman spectra was corrected using the software package Fityk.[8]

1.4. Synchrotron IR-spectroscopy

High-pressure synchrotron IR-spectroscopy was carried out at the National Synchrotron Light Source II (NSNL-II) in Upton, USA, at the Frontier Synchrotron Infrared Spectroscopy beamline 22-IR-1 (FIS). Fourier transform infrared (FTIR) measurements in a DAC were performed in transmission mode using a Bruker Vertex 80v FTIR spectrometer and a custom IR microscope system with a wide-band MCT detector in the range of 400–5000 cm^{-1} (≈ 1 cm^{-1} spectral sampling). The spot size on the sample was adjusted to be ≈ 20 µm.

*d.spahr@kristall.uni-frankfurt.de

1.5. Single-crystal synchrotron X-ray diffraction

High-pressure single-crystal synchrotron X-ray diffraction was carried out at the Advanced Photon Source (APS) in Lemont, USA, at the high pressure X-ray diffraction beamline 13-ID-D (GSECARS). The beam size on the sample was $\approx 1.5 \times 1.5$ µm^2. The diffraction data were collected using an Eiger2 X 9M CdTe detector, a wavelength of 0.3344 Å (37 keV) and a detector to sample distance of 187 mm. We rotated the DAC by $\pm 33°$ around the vertical axis perpendicular to the beam while collecting frames in 0.2° steps with 3 s acquisition time per frame.

The detector to sample distance was calibrated using the powder diffraction pattern of a LaB_6 standard in conjunction with the software DIOPTAS.[9] The diffractometer/detector geometry for the analysis of the single-crystal diffraction data was calibrated using diffraction data collected from a silicon single-crystal at ambient pressure. After the data collection, the reflections were indexed and integrated employing CrysAlis^{PRO} (version 43.67a).[10] We used the Domain Auto Finder program (DAFi) to find possible single-crystal domains for the subsequent data reduction.[11] The structure solution and refinement were performed using the software package OLEX2 employing SHELXT for the crystal structure determination and SHELXL for the refinement.[12,13,14] XRD maps were measured on a grid with a step-size of 3 µm and the software XDI was employed for the visualization of the diffraction map.[15]

1.6. Density functional theory-based calculations

First-principles calculations were carried out within the framework of density functional theory (DFT), employing the Perdew-Burke-Ernzerhof (PBE) and the PBEsol exchange-correlation functional and the plane wave/pseudopotential approach implemented in the CASTEP simulation package.[16,17,18,19] CASTEP and auxiliary programs, including those for a symmetry analysis, an analysis of the electron density distribution, and for visualizations, were run within the BIOVIA Materials Studio suite of programs.[20]

"On the fly" norm-conserving or ultrasoft pseudopotentials generated using the descriptors in the CASTEP data base were employed in conjunction with plane waves up to a kinetic energy cutoff of 1020 eV or 630 eV, for norm-conserving and ultrasoft pseudopotentials, respectively. The accuracy of the pseudopotentials is well established.[21] A correction scheme for van der Waals (v.d.W.) interactions was applied in the DFT-calculations with the PBE exchange-correlation functional. We employed the correction scheme developed by Tkatchenko and Scheffler.[22] A Monkhorst-Pack grid was used for Brillouin zone integrations.[23] We used a distance between grid points of $<$0.023 Å^{-1}. Convergence criteria for geometry optimization included an energy change of $<5 \times 10^{-6}$ eV atom^{-1} between steps, a maximal force of $<$0.008 eV Å^{-1} and a maximal component of the stress tensor $<$0.02 GPa. Phonon frequencies were obtained from density functional perturbation theory (DFPT) calculations.[24,25] Raman intensities were computed using DFPT with the "$2n + 1$" theorem approach.[26]

2. Results

2.1. *Single-crystal synchrotron X-ray diffraction*

We employed synchrotron X-ray diffraction in order to determine the crystal structure of the unknown phase present after laser-heating (9(1) GPa). First, we collected X-ray diffraction data on a grid across the sample chamber using a spot size of approximately 1.5×1.5 µm^2. In the second step, we collected diffraction data suitable for single-crystal X-ray diffraction analysis on selected locations, where unknown reflections occur in the X-ray diffraction map (at $2\theta \approx 6.03°$), which cannot be assigned to known phases. Fig. S 1 a shows a reciprocal space reconstruction for the ($h1k$) and the ($1kl$) planes (*unwarped*-image) after data processing in CrysAlis on the location selected for the single-crystal structure solution.

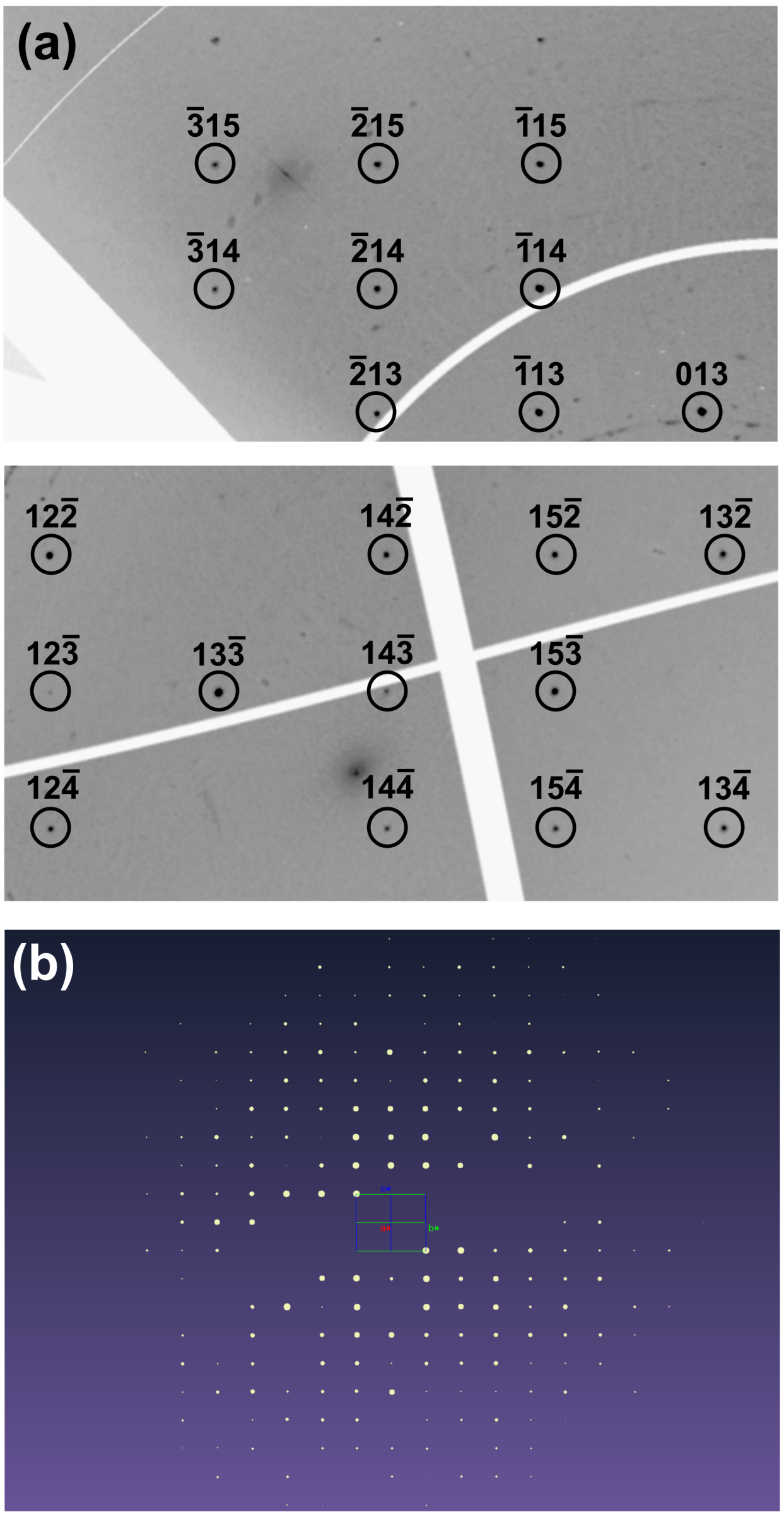

Figure S 1: (a) Reciprocal space reconstruction for the ($h1k$) plane (top) and the ($1kl$) plane (bottom). (b) Schematic depiction of the reflections in reciprocal space using the Ewald-Explorer in CrysAlis after data reduction which were later used for the refinement. The projection is shown along a^*.

In addition to the reflection of the unknown phase, we observed reflections and powder rings of CO_2-I (dry-ice), H_2CO_3-$P2_1/n$, H_2O-VII and of the diamonds in the diffraction map collected after the synthesis.[27,28,29] At the position of the structure determination of the yet unknown phase, diffraction peaks of H_2CO_3-$P2_1/n$ were not observed. Fig. S 1 b shows the projection of the reciprocal space along a^*. The effect of the shading of diffracted beams due to the metallic body of the DAC and the effect of the inactive detector-areas due to the segments of the Eiger2 X detector can be observed (Fig. S 1).

Table S 1: Structural parameters of H_2CO_3-*Pnma* at 9(1) GPa from synchrotron single-crystal structure solution (ambient temperature). Data derived from DFT calculations are shown for comparison (athermal limit).

	Single Crystal	DFT
**Crystal data**		
Crystal system	orthorhombic	orthorhombic
Space group	*Pnma*	*Pnma*
Chemical formula	H_2CO_3	H_2CO_3
M_r	62.03	62.03
a (Å)	4.994(5)	4.9820
b (Å)	6.547(2)	6.3860
c (Å)	5.330(2)	5.1956
α (°)	90.0	90.0
β (°)	90.0	90.0
γ (°)	90.0	90.0
V (Å^3)	174.2(2)	165.30
Z	4	4
**Data collection**		
F_{000}	128	-
θ range (°)	2.93–20.53	-
measured reflections	608	-
independent reflections	326	-
reflections $I > 2\sigma(I)$	256	-
R_{int}	0.012	-
**Refinement**		
$R_1[I > 2\sigma(I)]$, $wR_2(I)$	0.044, 0.107	-
No. of reflections	326	-
No. of parameters	26	-
No. of restraints	0	-
No. of constraints	0	-
$\Delta\rho_{max}$, $\Delta\rho_{min}$ (e Å^{-3})	0.32, -0.34	-

We solved the crystal structure of the unknown phase in the orthorhombic space group *Pnma* (No. 64) with $Z = 4$. The chemical composition of the unknown phase is H_2CO_3. The R_1-value (4.4%) of the refinement is reasonable low for a DAC experiment, while the reflection to parameter ratio (12.5:1) is high. The displacement parameters of the carbon and the oxygen atoms are refined anisotropically. The displacement parameter of the hydrogen atom was refined isotropically. No constraints or restraints had to be introduced for a stable structure refinement. The experimental crystallographic parameters of H_2CO_3-*Pnma* at 9(1) GPa are listed in Table S 1 in com-

parison to structural data derived from our DFT-based geometry optimization at the same pressure. Our structural data for are in good agreement with the predicted structural model for H_2CO_3-*Pnma* suggested in an earlier computational study as the stable phase in the pressure range between 1 GPa and 44 GPa.[30]

2.2. Synchrotron IR-spectroscopy

We measured synchrotron-based IR spectroscopy in the sample chamber of the DAC, which contains H_2CO_3-*Pnma* as well as H_2CO_3-$P2_1/n$ at 9(1) GPa using a spot size on the sample of ≈ 20 µm. In a small area of the sample chamber, where no reaction had occurred, we could observe the characteristic peaks corresponding to CO_2 and to H_2O and no contribution of a H_2CO_3 phase (Fig. S 2).[31,32] The experimental IR data of CO_2-I are in agreement wit our DFT-based calculations. In the laser heated area we observed the IR-signature of H_2CO_3 in a spectral region between 1200 cm^{-1} and 2000 cm^{-1} (Fig. S 2). Our experimental spectra are in agreement with our DFT-based calculations for both phases. The difference between H_2CO_3-*Pnma* and H_2CO_3-$P2_1/n$ could not be resolved in IR spectra collected at different positions in the sample chamber.

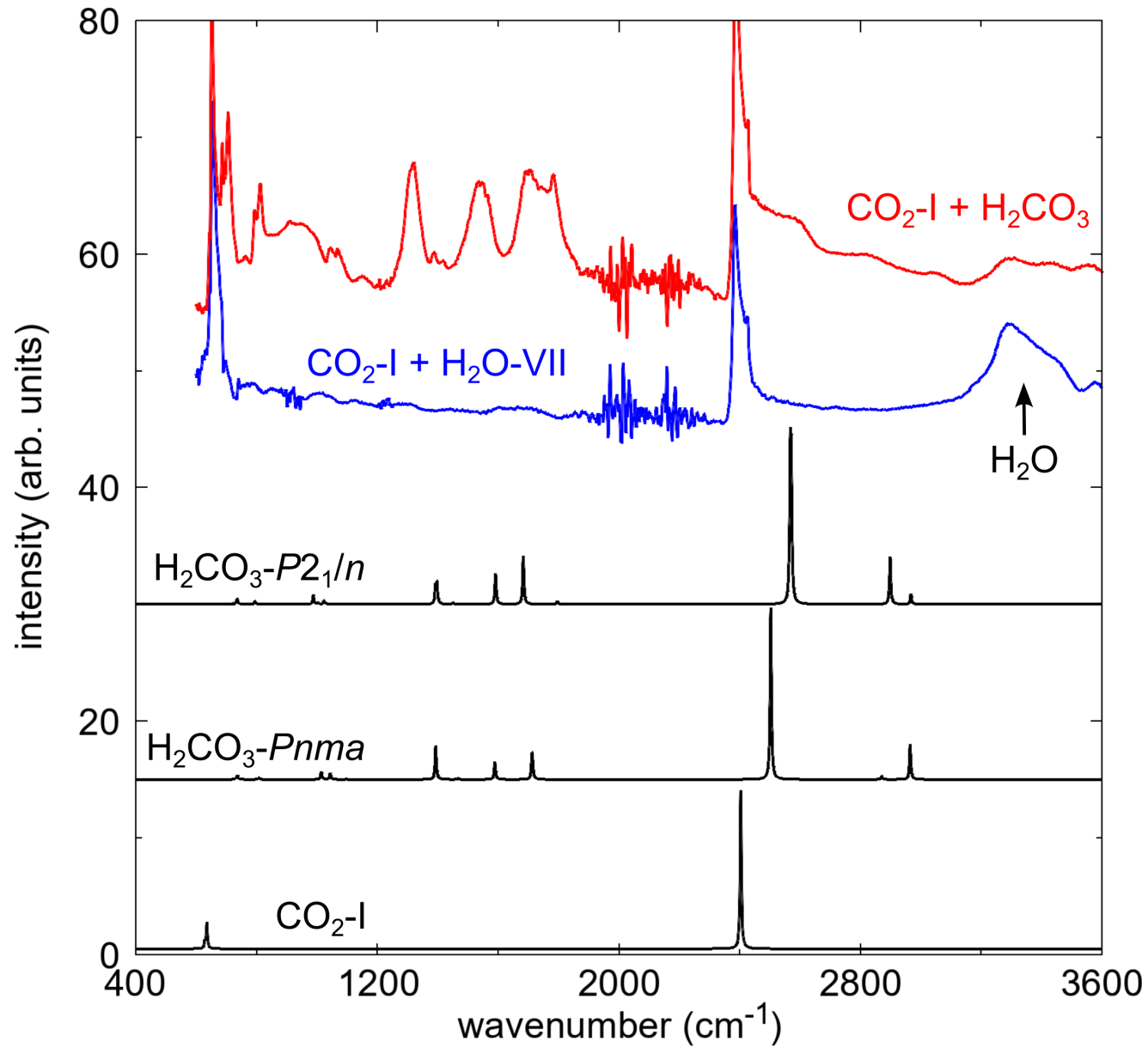

Figure S 2: Theoretical IR spectra for CO_2-I, H_2CO_3-*Pnma* and H_2CO_3-$P2_1/n$ derived from the DFT-based calculations at 9 GPa. Experimental synchrotron IR spectra of an unheated CO_2 + H_2O mixture and a H_2CO_3-*Pnma* + H_2CO_3-$P2_1/n$ mixture in the heated area of the sample chamber. The spectra were measured in the same DAC at 9(1) GPa.

2.3. Compressibility of H_2CO_3-*Pnma*

We used our DFT-based calculations to obtain the p, V relation for H_2CO_3-*Pnma* in the pressure range between 0 GPa and 20 GPa. We fitted a 3rd-order Birch-Murnaghan equation of states (EoS) to the theoretical p, V-data using the software package EOSFit7-GUI in order to determine the bulk modulus and its derivative for H_2CO_3-*Pnma* (Fig. S 3).[33,34,35] From the EoS fit to the theoretical data in the pressure range between 0 GPa and 20 GPa we obtained a bulk modulus of $K_0 = 12.3(3)$ GPa with $K_p = 5.8(1)$ for H_2CO_3-*Pnma*. Within the expected uncertainties our data match with the values derived for H_2CO_3-$P2_1/n$ ($K_0 = 14.2(4)$ GPa with $K_p = 6.1(1)$) obtained in a similar manner.[27]

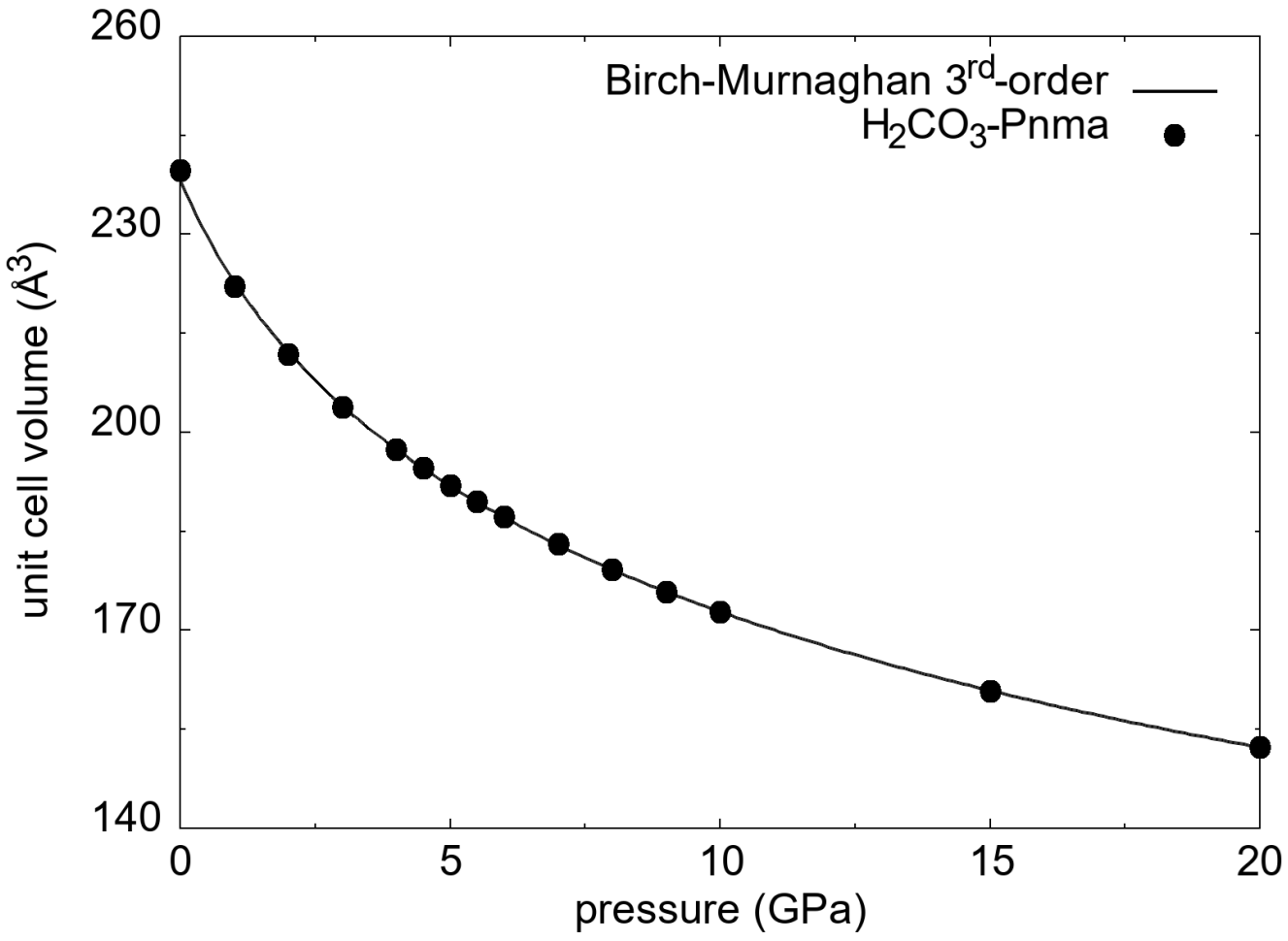

Figure S 3: A Birch-Murnaghan EoS was fitted to the theoretical p, V-data of H_2CO_3-*Pnma* obtained by DFT-based calculations between 0–20 GPa.

References

(1) Boehler, R. New diamond cell for single–crystal X-ray diffraction. *Rev. Sci. Instrum.* **2006**, *77*, 115103–1–115103–3, DOI: 10.1029/JB091iB05p04673

(2) Mao, H. K.; Xu, J.; Bell, P. M. Calibration of the ruby pressure gauge to 800 kbar under quasi-hydrostatic conditions. *J. Geophys. Res.* **1986**, *91*, 4673–4676, DOI: 10.1029/JB091iB05p04673

(3) Spahr, D.; Bayarjargal, L.; Brüning, L.; Kovalev, V.; Bykova, E.; Bykov, M.; Milman, V.; Mezouar, M.; Winkler, B. Synthesis and Crystal Structure of Anhydrous Di-iodyl Carbonate $(IO_2)_2[CO_3]$, Hosting I^{5+}-Cations. *JACS Au* **2025**, *5*, 4675–4680, DOI: 10.1021/jacsau.5c00829

(4) Bayarjargal, L.; Fruhner, C.-J.; Schrodt, N.; Winkler, B. $CaCO_3$ phase diagram studied with Raman spectroscopy at pressures up to 50 GPa and high temperatures and DFT modeling. *Phys. Earth Planet. Inter.* **2018**, *281*, 31–45, DOI: 10.1016/j.pepi.2018.05.002

(5) Draper, J. W. On the production of light by heat. *Lond. Edinb. Dublin Philos. Mag. J. Sci.* **1847**, *30*, 345–360, DOI: 10.1080/14786444708647190

(6) Benedetti, L. R.; Loubeyre, P. Temperature gradients, wavelength-dependent emissivity, and accuracy of high and very-high temperatures measured in the laser-heated diamond cell. *High Press. Res.* **2004**, *24*, 423–455, DOI: 10.1080/08957950412331331718

(7) Du, Z.; Amulele, G.; Benedetti, L. R.; Lee, K. K. M. Mapping temperatures and temperature gradients during flash heating in a diamond-anvil cell. *Rev. Sci. Instrum.* **2013**, *84*, 075111, DOI: 10.1063/1.4813704

(8) Wojdyr, M. *Fityk*: a general-purpose peak fitting program. *J. Appl. Cryst.* **2010**, *43*, 1126–1128, DOI: 10.1107/S0021889810030499

(9) Prescher, C.; Prakapenka, V. B. *DIOPTAS*: a program for reduction of two-dimensional X-ray diffraction data and data exploration. *High. Press. Res.* **2015**, *35*, 223–230, DOI: 10.1080/08957959.2015.1059835

(10) Agilent, CrysAlis PRO, Yarnton, England, **2014**

(11) Aslandukov, A.; Aslandukov, M.; Dubrovinskaia, N.; Dubrovinsky, L. *Domain Auto Finder* (*DAFi*) program: the analysis of single-crystal X-ray diffraction data from polycrystalline sample. *J. Appl. Cryst.* **2022**, *55*, 1383–1391, DOI: 10.1107/S1600576722008081

(12) Dolomanov, O. V.; Bourhis, L. J.; Gildea, R. J.; Howard, J. A. K.; Puschmann, H. *OLEX2*: a complete structure solution, refinement and analysis program. *J. Appl. Cryst.* **2009**, *42*, 339–341, DOI: 10.1107/S0021889808042726

(13) Sheldrick, G. M. *SHELXT* — Integrated space-group and crystal-structure determination. *Acta. Cryst.* **2015**, *A71*, 3–8, DOI: 10.1107/S2053273314026370

(14) Sheldrick, G. M. Crystal structure refinement with *SHELXL*. *Acta. Cryst.* **2015**, *C71*, 3–8, DOI: 10.1107/S2053229614024218

(15) Hrubiak, R.; Smith, J. S.; Shen, G. Multimode scanning X-ray diffraction microscopy for diamond anvil cell experiments. *Rev. Sci. Instrum.* **2019**, *90*, 025109, DOI: 10.1063/1.5057518

(16) Hohenberg, P.; Kohn, W. Inhomogeneous Electron Gas. *Phys. Rev.* **1967**, *136*, B864–B871, DOI: 10.1103/PhysRev.136.B864

(17) Perdew, J. P.; Burke, K.; Ernzerhof, M. Generalized Gradient Approximation Made Simple. *Phys. Rev. Lett.* **1996**, *77*, 3865–3868, DOI: 10.1103/PhysRevLett.77.3865

(18) Clark, S. J.; Segall, M. D.; Pickard, C. J.; Hasnip, P. J.; Probert, M. I. J.; Refson, K.; Payne, M. C. First principles methods using CASTEP. *Z. Kristallogr.* **2005**, *220*, 567–570, DOI: 10.1524/zkri.220.5.567.65075

(19) Perdew, J. P.; Ruzsinszky, A.; Csonka, G. I.; Vydrov, O. A.; Scuseria, G. E.; Constantin, L. A.; Zhou, X.; Burke, K. Restoring the Density-Gradient Expansion for Exchange in Solids and Surfaces. *Phys. Rev. Lett.* **2008**, *100*, 136406, DOI: 10.1103/PhysRevLett.100.136406

(20) BIOVIA, Materials Studio 2025, San Diego, USA

(21) Lejaeghere, K.; Bihlmayer, G.; Björkman, T.; Blaha, P.; Blügel, S.; Blum, V.; Caliste, D.; Castelli, I. E.; Clark, S. J.; Dal Corso, A. et al. Reproducibility in density functional theory calculations of solids. *Science* **2016**, *351*, aad3000, DOI: 10.1126/science.aad3000

(22) Tkatchenko, A.; Scheffler, M. Accurate Molecular Van Der Waals Interactions from Ground-State Electron Density and Free-Atom Reference Data. *Phys. Rev. Lett.* **2009**, *102*, 073005, DOI: 10.1103/PhysRevLett.102.073005

(23) Monkhorst, H. J.; Pack, J. D. Special points for Brillouin-zone integrations. *Phys. Rev. B* **1976**, *13*, 5188–5192, DOI: 10.1103/PhysRevB.13.5188

(24) Baroni, S.; de Gironcoli, S.; Dal Corso, A.; Giannozzi, P. Phonons and related crystal properties from density-functional perturbation theory. *Rev. Mod. Phys.* **2001**, *73*, 515–562, DOI: 10.1103/RevModPhys.73.515

(25) Refson, K.; Tulip, P. R.; Clark, S. J. Variational density-functional perturbation theory for dielectrics and lattice dynamics. *Phys. Rev. B* **2006**, *73*, 155114, DOI: 10.1103/PhysRevB.73.155114

(26) Miwa, K. Prediction of Raman spectra with ultrasoft pseudopotentials. *Phys. Rev. B* **2011**, *84*, 094304, DOI: 10.1103/PhysRevB.84.094304

(27) Spahr, D.; Bykova, E.; Bayarjargal, L.; Bykov, M.; Brüning, L.; Kovalev, V.; Milman, V.; Giordano, N.; Liermann, H.-P.; Winkler, B. Crystal Structure of Carbonic Acid (H_2CO_3) at Elevated Pressures from

Single Crystal Diffraction. *Chem. Eur. J.* **2025**, *31*, e202501964, DOI: 10.1002/chem.202501964

(28) Simon, A.; Peters, K. Single-Crystal Refinement of the Structure of Carbon Dioxide. *Acta Cryst.* **1980**, *B36*, 2750–2751, DOI: 10.1107/S0567740880009879

(29) Gajda, R.; Chodkiewicz, M.; Zhang, D.; Nguyen, P.; Prakapenka, V.; Wozniak, K. Structure of ice VII with Hirshfeld atom refinement. *IUCrJ* **2025**, *12*, 288–294, DOI: 10.1107/S2052252525002581

(30) Saleh, G.; Oganov, A. R. Novel Stable Compounds in the C–H–O Ternary System at High Pressure. *Sci. Rep.* **2016**, *6*, 32486, DOI: 10.1038/srep32486

(31) Poteet, C. A.; Pontoppidan, K. M.; Megeath, S. T.; Watson, D. M.; Isokoski, K.; Bjorkman, J. E.; Sheehan, P. D.; Linnartz, H. Infrared observation of the phase transitions of ice at low temperatures and pressures up to 50 GPa and the metastability of low-temperature ice VII. *ApJ* **2013**, *766*, 117, DOI: 10.1088/0004-637X/766/2/117

(32) Song, M.; Yamawaki, H.; Fujihisa, H.; Sakashita, M.; Aoki, K. Infrared observation of the phase transitions of ice at low temperatures and pressures up to 50 GPa and the metastability of low-temperature ice VII. *Phys. Rev. B* **2003**, *68*, 024108, DOI: 10.1103/PhysRevB.68.024108

(33) Murnaghan, F. The Compressibility of Media under Extreme Pressures. *Proc. Natl. Acad. Sci.* **1944**, *30*, 244–247, DOI: 10.1073/pnas.30.9.244

(34) Birch, F. Finite Elastic Strain of Cubic Crystals. *Phys. Rev.* **1947**, *71*, 809–824, DOI: 10.1103/PhysRev.71.809

(35) Gonzalez-Platas, J.; Alvaro, M.; Nestola, F.; Angel, R. *EosFit7-GUI*: a new graphical user interface for equation of state calculations, analyses and teaching. *J. Appl. Cryst.* **2016**, *49*, 1377–1382, DOI: 10.1107/S1600576716008050
